# Flipping the “switch” on mutant p53 by zinc metallochaperones: how a brief pulse of zinc can reactivate mutant p53 to kill cancer

**DOI:** 10.18632/oncotarget.26561

**Published:** 2019-01-29

**Authors:** Xin Yu, Darren R. Carpizo

**Affiliations:** Darren R. Carpizo: Department of Surgery, Rutgers Robert Wood Johnson Medical School, New Brunswick, NJ, USA; Rutgers Cancer Institute of New Jersey, New Brunswick, NJ, USA; Department of Pharmacology, Rutgers University, Piscataway, NJ, USA; Z53 Therapeutics Inc

**Keywords:** mutant p53, zinc metallochaperones, switch

The majority of cancer is driven by mutations in the *TP53* gene and the majority of these mutations are missense creating a large variety of mutant proteins found at high levels in cancer cells [1]. Based on this, restoring wild type function to these proteins using a small molecule has been a high priority in cancer therapeutics. In several ways, mutant p53 is similar to any oncogene in its attractiveness as a therapeutic target as 1) it is not expressed in non-cancerous tissue, 2) it is overexpressed in the cancer cell and 3) the biological effect of restoring its function is significant. But, how long do you need wild type p53 to be “turned on” to achieve the desired effect (p53 mediated cell death)? The traditional paradigm in targeted cancer drug development is to develop compounds that will bind to their target as tightly as possible and to select for a pharmacology that will drive exposure through both dose and pharmacokinetics. In other words, we want our traditional targeted therapeutic to be “on” all the time, or as much as possible. Clinical trial dosing schedules are usually determined by the maximum tolerated dose rather than the dose necessary to achieve the biological effect on the target. This often leads to unnecessary toxicity. However, we have recently learned that with p53, this is not necessary. Only a brief period (<30 minutes) of wild type p53 function is needed to achieve cancer cell death [2]. This research has allowed us to appreciate that mutant p53 is a distinctive target in that the duration of function required for therapeutic benefit is far less than that of a drug targeting a typical oncogene.

Since the mid 1990’s when the x-ray crystal structure of the p53 DNA binding domain was solved we have learned that p53 requires the binding of zinc for structural purposes, and that by starving p53 of zinc, the protein will misfold and amazingly it will refold properly when zinc is given back [3-5]. This idea that the structure of p53 is malleable by manipulating zinc forms the basis for zinc metallochaperone therapy. Through biochemical and biophysical research of various p53 missense mutants we have learned that cancer exploits the relationship between zinc and p53 in that a number of substituted residues weaken the protein’s affinity for zinc. The best example of this is the most common missense mutant in cancer, p53^R175H^ [6]. Our group and in particular the laboratory of Dr. Stewart Loh was the first to directly measure the affinity of the p53^R175H^ mutant for zinc indicating that the mutant bound zinc at least 1000 fold less tightly [7]. This means that raising intracellular zinc levels approximately 1000 fold would in theory allow zinc to bind to the p53^R175H^ and this would induce a wild type conformation change. We initially discovered a set of thiosemicarbazone metal ion chelators that could perform this function and we have named these zinc metallochaperones (ZMCs) [7-9]. ZMCs are small molecules that bind zinc in 2:1 molar ratio and pass through the plasma membrane functioning as a zinc ionophore to raise intracellular zinc levels [10]. These compounds are soft chelators such that their affinity for zinc is tight enough to steal zinc from plasma binding proteins but weak enough to donate it to mutant p53.

We initially observed in cells treated with a ZMC, the mutant reactivating function was transient over a 24 hour period. Specifically, the function would come on within 6-8 hours and off by 24 hours. In Yu and Kogan et al. we now have learned that this transient activity is due to the cell’s response to this surge in zinc [2]. Mammalian cells have several families of genes that function to maintain cellular free zinc levels exquisitely tight. These zinc homeostatic mechanisms normalize zinc levels in response to ZMC therapy which results in zinc no longer binding to mutant p53 and the function of the drug is off. Once understood, we hypothesized that the duration of exposure to a ZMC need not be continuous (certainly not longer than 6 hours). Through a series of washout experiments we found that only a brief period of an exposure (≤30 minutes) was sufficient to result in massive cancer cell death. We then translated these findings *in vivo* by showing that ZMC1, a compound with a half-life of only 30 minutes was capable of more than doubling the median survival of mice from a genetically engineered murine pancreatic cancer model harboring a zinc deficient (p53^R172H^) allele while having no effect in mice with a non-zinc deficient allele (p53^R270H^). This proof-of-concept pre-clinical data serves to substantiate the claim that zinc metallochaperone therapy is a viable strategy to reactivate mutant p53 for drug development. Importantly, this also indicates that the patient population for ZMC’s is defined by those with zinc deficient p53 mutations. In drug development, knowing who will benefit (or who will not) from an investigational agent is a considerable advantage.

The paper by Yu and Kogan et al. is also significant for the demonstration of a novel ZMC - zinc complex in which the drug is synthesized in a 2:1 molar ratio with zinc. This idea was based on the x-ray crystal structure of ZMC1 bound to zinc indicating that two molecules of ZMC1 bound one molecule of zinc [10]. Here the authors show that these zinc loaded complexes are more effective than their monomers both *in vitro* and *in vivo*. This indicates that the future ZMC that makes it to the clinic will most likely be synthesized in complex with zinc. The “switch” concept for the function of ZMCs is unique in cancer drug development (Figure 1). This knowledge indicates that this therapy should be viewed as a pulsatile type therapy and therefore the clinical candidate ZMC should not be developed using the traditional paradigm for targeted drug development (i.e. maximal half-life, maximal exposure), but rather a short half-life and a minimum concentration in the blood needed to achieve sufficient zinc delivery.

**Figure 1:**
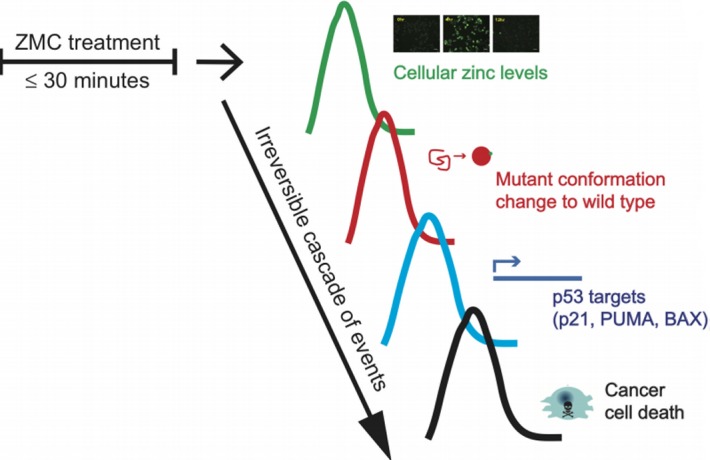
The “switch” concept for the function of ZMCs in cancer drug development With as little as 30 minutes of ZMC therapy, the switch is turned ON and an irreversible cascade of events is initiated in a cancer cell expressing a zinc deficient missense mutant p53. First, free zinc levels rise, then zinc becomes bound to the mutant p53 and induces a wild type conformation. This results in the induction of a wild type p53 mediated apoptotic program that results in cancer cell death. Cellular zinc homeostatic mechanisms then normalize the zinc levels, zinc is no longer bound to mutant p53 and the drug is OFF.

## References

[1] Blanden AR (2015). Drug Discov Today.

[2] Yu X (2018). Clin Cancer Res.

[3] Cho Y (1994). Science.

[4] Meplan C (2000). Oncogene.

[5] Hainaut P (2001). Antioxid Redox Signal.

[6] Freed-Pastor WA (2012). Genes Dev.

[7] Yu X (2014). Oncotarget.

[8] Yu X (2012). Cancer Cell.

[9] Yu X (2017). Mol Pharmacol.

[10] Blanden AR (2015). Mol Pharmacol.

